# Incidence, clinical presentation, and antimicrobial resistance trends in *Salmonella* and *Shigella* infections from children in Yucatan, Mexico

**DOI:** 10.3389/fmicb.2013.00288

**Published:** 2013-10-01

**Authors:** Mussaret B. Zaidi, Teresa Estrada-García, Freddy D. Campos, Rodolfo Chim, Francisco Arjona, Magda Leon, Alba Michell, Damien Chaussabel

**Affiliations:** ^1^Microbiology Research Laboratory, Hospital General O’Horan, MéridaYucatán, Mexico; ^2^Infectious Diseases Research Unit, Hospital Regional de Alta Especialidad de la Penïnsula de YucatánMerida, Mexico; ^3^Department of Molecular Biomedicine, Centro de Investigación y de Estudios Avanzados del Instituto Politécnico NacionalMexico City, Mexico; ^4^Benaroya Research InstituteSeattle, WA, USA

**Keywords:** *Salmonella*, *Shigella*, incidence, antimicrobial resistance, beta-lactamase genes, *bla*_CMY-2_, *qnr* genes, Mexico

## Abstract

**Background:** *Salmonella* and *Shigella* cause significant morbidity and mortality among children worldwide. Increased antimicrobial resistance results in greater burden of disease.

**Materials and Methods:** From 2005 to 2011, *Salmonella* and *Shigella* isolates collected from ill children at a major hospital in Yucatan, Mexico, were subjected to serotyping and antimicrobial susceptibility testing by disk diffusion and agar dilution. The identification of *bla*_CTX_, *bla*_CMY_, *bla*_SHV_, *bla*_TEM_, and *bla*_OXA_ and *qnr* resistance genes was conducted by PCR and sequencing.

**Results:** Among 2344 children with acute gastroenteritis, salmonellosis decreased from 17.7% in 2005 to 11.2% in 2011 (*p* < 0.001). In contrast, shigellosis increased from 8.3% in 2010 to 12.1% in 2011. Compared to children with *Salmonella*, those with *Shigella* had significantly more bloody stools (59 vs 36%, *p* < 0.001), dehydration (27 vs 15%, *p* = 0.031), and seizures (11 vs 3%, *p* = 0.03). In *Salmonella* (*n* = 365), there was a significant decrease in resistance to ampicillin (43 to 16%, *p* < 0.001), trimethoprim–sulfamethoxazole (44 to 26%, *p* = 0.014), and extended-spectrum cephalosporins (27 to 10%, *p* = 0.009). Reduced susceptibility to ciprofloxacin in *Salmonella* rose from 30 to 41% (*p* < 0.001). All ceftriaxone-resistant isolates harbored the *bla*_CMY-2_ gene. *qnr* genes were found in 42 (36%) of the 117 *Salmonella* isolates with a ciprofloxacin MIC ≥ 0.125 μg/ml. Four were *qnrA1* and 38 were *qnrB19*. Resistance to ampicillin (40%) and trimethoprim–sulfamethoxazole (58%) was common in *Shigella* (*n* = 218), but isolates remained fully susceptible to ceftriaxone and ciprofloxacin.

**Conclusion:** Illness from *Salmonella* has decreased while severe *Shigella* infections have increased among children with gastroenteritis in the Yucatan Peninsula. While *Shigella* resistance to clinically important antibiotics remained unchanged, resistance to most of these, except ciprofloxacin, declined in *Salmonella*. *bla*_CMY-2_ and *qnr* genes are common in *Salmonella* isolates.

## INTRODUCTION

*Salmonella* and *Shigella* are associated with a high burden of illness among children in the developing world (Niyogi, 2005; Woc-Colburn and Bobak, 2009; Kotloff et al., 2013). In Mexico, the disease patterns of these pathogens have undergone significant changes in the last decades. During the early 1970s, *Shigella dysenteriae* type 1 and *Salmonella* Typhi caused severe outbreaks among the local population. The epidemic strains of *S*. Typhi and *S*. *dysenteriae* were both multidrug resistant (MDR) to chloramphenicol, tetracycline, streptomycin, and sulfonamides, harbored on different plasmids (Datta and Olarte, 1974). Subsequently, *S*.* dysenteriae* infections decreased, while *Shigella flexneri* became the predominant species isolated from endemic infections. *S*.* dysenteriae*, nonetheless, was still prevalent among visitors to the Yucatan Peninsula during the late 1980s (Centers for Disease Control [CDC], 1988). Typhoid fever also sharply declined, while non-typhoidal *Salmonella*, extremely prevalent along the food chain, was identified as one of the main causes of gastroenteritis in hospitalized children. *Salmonella* Typhimurium, commonly carrying resistance to ampicillin, chloramphenicol, streptomycin, sulfonamides, and tetracycline, was the most common serotype encountered in Yucatan (Zaidi et al., 2006). Extended-spectrum cephalosporin (ESC)-resistant *S*. Typhimurium isolates first emerged in 2002, and rapidly spread throughout the state causing severe diarrhea and fatal systemic infections in infants (Zaidi et al., 2007).

In the past 25 years, the combination of the widespread use of oral rehydration solutions, rising antimicrobial resistance, and regional socioeconomic changes has modified the epidemiology and the impact of these enteric pathogens (von Seidlein et al., 2006; Parry and Threlfall, 2008). There is a clear need to update relevant epidemiologic data (Sansonetti, 2006) as there are few studies from Latin America. Monitoring for ESC and fluoroquinolone resistance is particularly important because these compounds are among the few therapeutic options available for severe *Salmonella* and *Shigella* infections. In this study, the main objectives were (1) to analyze trends in incidence and antimicrobial resistance of *Salmonella* and *Shigella* infections in children with gastroenteritis and/or systemic infections who sought medical attention at a major hospital in the Yucatan Peninsula, (2) to compare the clinical presentations of the ill children, and (3) determine the prevalence of extended-spectrum beta-lactamases, CMY-2 and Qnr in *Salmonella* and *Shigella* isolates.

## MATERIALS AND METHODS

### SETTING

The Hospital General O’Horan is a tertiary-care public hospital that receives patients from throughout the state of Yucatan and neighboring states in southeast Mexico. The Pediatrics Emergency Department has an active surveillance program for diarrheal pathogens, current since 2000, which includes children with mild to severe gastroenteritis as well as those with systemic infections. Stool cultures are systematically collected from children with diarrhea. Blood cultures are also routinely collected from children who appear septic or develop hematological, neurological, or other extraintestinal complications.

### CLINICAL AND EPIDEMIOLOGICAL DATA

A trained nurse administered standardized questionnaires to collect demographic and clinical information on the childrens’diarrheal episodes.

### MICROBIOLOGY

Stool samples were collected in sterile containers containing Cary–Blair media. Samples were inoculated onto XLD, Hektoen Enteric, and brilliant green agars as well as tetrathionate and Rappaport broths and incubated at 35°C for 18–24 h. Broths were subcultured to XLD and brilliant green agar plates and incubated for another 18–24 h. Identification of *Salmonella* and *Shigella* isolates were performed with conventional biochemical tests. *Salmonella* isolates were serotyped by the Kauffman–White scheme using commercial antisera (Becton Dickinson, Franklin Lakes, NJ, USA). All *Shigella* isolates were serogrouped; serotyping was performed on *S. dysenteriae* isolates only. Antimicrobial susceptibility testing was routinely performed for ampicillin, chloramphenicol, gentamicin, nalidixic acid, trimethoprim–sulfamethoxazole, ciprofloxacin, ceftriaxone, ceftazidime, and tetracycline by disk diffusion (CLSI, 2012a). Minimum inhibitory concentrations (MICs) for ciprofloxacin, azithromycin, ceftriaxone, nalidixic acid, and furazolidone were determined by agar dilution (CLSI, 2012b). Susceptibility and resistance breakpoints used for azithromycin were 16 and 32 μg/ml, respectively (Sjolund-Karlsson et al., 2011) and 2 μg/ml and 8 μg/ml for furazolidone (Rampling et al., 1990). Ceftriaxone and/or ceftazidime non-susceptible isolates were tested on a secondary panel of beta-lactam antimicrobials that included cefepime, cefotaxime, cefoxitin, imipenem, meropenem, amoxicillin–clavulanic acid, and piperacillin-tazobactam. According to resistance phenotypes, these isolates were also screened for AmpC beta-lactamase production with a combination disk method using cefoxitin (30 μg) and cefoxitin with boronic acid (30 μg + 300 μg; Jacoby et al., 2006).

### GENETIC CHARACTERIZATION OF *bla* and *qnr* GENES

All ceftriaxone-resistant isolates (MIC ≥ 4) were tested for the presence of beta-lactamase genes. A multiplex PCR was used for the detection of genes encoding the CTX, SHV, TEM, and OXA enzymes according to previously published methods (Briñas et al., 2002; Edelstein et al., 2003). Detection of the *bla*_CMY_ gene was performed using a previously described assay (Zhao et al., 2001). *Salmonella* isolates with ciprofloxacin MICs ≥ 0.125 μg/ml were tested for *qnrA*, *qnrB*, and *qnrS* genes following published methods (Robicsek et al., 2006).

Specific group primers used for the study are listed in **Table 1**. Control strains were kindly provided by Patrick McDermott (U.S. Food and Drug Administration) and Jesus Silva-Sanchez (Instituto Nacional de Salud Publica, Mexico).

**Table 1:** Specific group primers used for PCR assays for beta-lactamase and *qnr* genes.

PCR name	Primer name	Sequence (5′–3′)	Length (base)	Amplicon size (pb)
Multiplex TEM, SHV, CTX, OXA	tem-F	TTCTTGAAGACGAAAGGGC	19	1150
	tem-R	ACGCTCAGTGGAACGAAAC	19	
	shv-F	CACTCAAGGATGTATTGTG	19	702
	shv-R	TTAGCGTTGCCAGTGCTCG	19	
	ctx-F	TTTGCGATGTGCAGTACCAGTAA	23	544
	ctx-R	CGATATCGTTGGTGGTGCCATA	22	
	oxa-F	TTCAAGCCAAAGGCACGATAG	21	885
	oxa-R	TCCGAGTTGACTGCCGGGTTG	21	
CMY	cmy-F	GACAGCCTCTTTCTCCACA	19	1000
	cmy-R	TGGAACGAAGGCTACGTA	18	
Multiplex qnrA, qnrB and qnrS	qnrA-F	ATTTCTCACGCCAGGATTTG	20	516
	qnrA-R	GATCGGCAAAGGTTAGGTCA	20	
	qnrB-F	GATCGTGAAAGCCAGAAAGG	20	469
	qnrB-R	ACGATGCCTGGTAGTTGTCC	20	
	qnrS-F	ACGACATTCGTCAACTGCAA	20	417
	qnrS-R	TAAATTGGCACCCTGTAGGC	20	

Amplified products were separated by gel electrophoresis on 2% agarose and stained with ethidium bromide. Sequencing was performed using both forward and reverse PCR primers on a ABI Prism 3130 XL Genetic Analyzer (Applied Biosystems). Sequences were analyzed using the National Center for Biotechnology Information’s BLAST network service (). Sequences were compared to the original gene sequences reported in the GenBank database of accession numbers J01749, X91840, AY070235, EU432277, FJ460235 (Sutcliffe, 1979; Bauernfeind et al., 1996; Jacoby et al., 2008; Wiesner et al., 2009). Sequences for *qnrA1* and *qnrB19* were submitted under accession numbers KF517418 and KF517417, respectively.

### DATA ANALYSIS

The chi-square test was used to detect resistance trends over time and differences in clinical presentations for each pathogen; a *p* value < 0.05 was considered significant. The odds ratio and its 95% confidence interval (CI) were calculated for the different clinical variables. Antimicrobial susceptibility results were analyzed with Whonet software version 5.6 (www.whonet.org).

### ETHICS

The data was obtained from two different studies that were approved by the Hospital General O’Horan Research and Ethics Committee; written informed consent was obtained from the children’s guardians to collect stool samples and use the data for scientific purposes. For this analysis, clinical and microbiological data were entered on new databases; a unique numerical code was assigned to each patient and personal identifiers were removed to ensure anonymity.

## RESULTS

### EPIDEMIOLOGY

**FIGURE 1:**
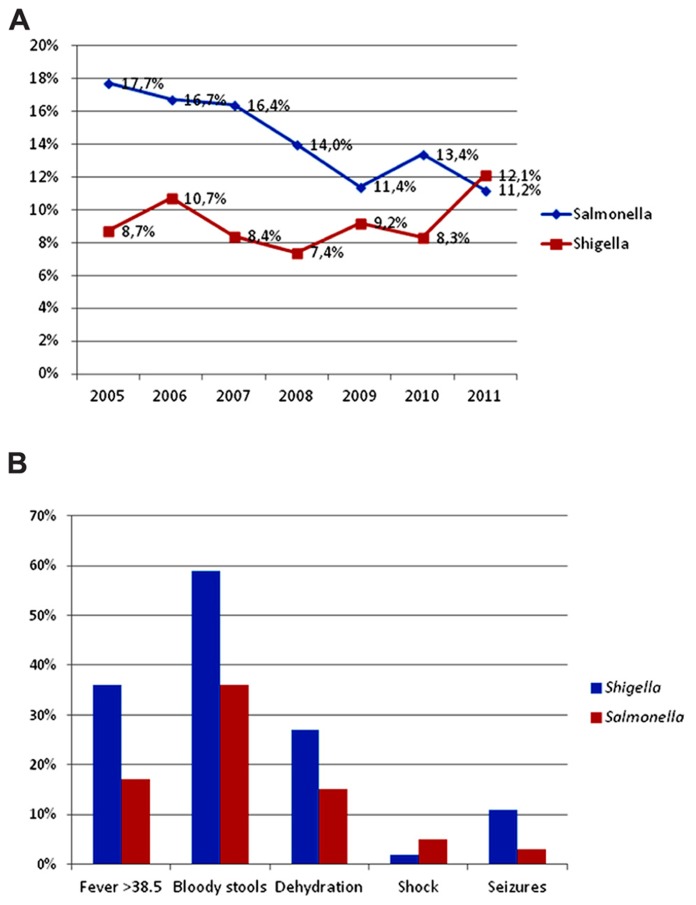
**Frequency and clinical presentation of *Salmonella* and *Shigella* infections in ill children at a state referral hospital inYucatan, Mexico, 2005–2011.** **(A)** There was a statistically significant trend toward decreasing *Salmonella* gastroenteritis, from 17.7% in 2005 to 11.2% in 2011 (*p* < 0.001). In contrast, there was a precipitous increase in shigellosis from 8.3% in 2010 to 12.1% in 2011. **(B)** Children with *Shigella* had twice as much fever ≥38.5°C, bloody stools, dehydration, and almost four times as many seizures, compared to those with *Salmonella*. Children with salmonellosis, however, were more likely to be admitted with hypovolemic shock.

From January 2005 to December 2011, 2344 children under 10 years of age who were admitted to the hospital for acute gastroenteritis were included in the study. One child with fever and malaise without gastroenteritis, whose blood culture was positive for *S*. Typhi, was also included. Children came from 94 localities within the state of Yucatan and from 35 other cities or towns in the neighboring states. Eight hundred ninety-one (38%) were under 1 year of age, 1176 (54%) were between 1 and 4 years of age, and 186 (8%) were between 5 and 9 years of age. *Salmonella* gastroenteritis decreased from 17.7% of all cases in 2005 to 11.2% in 2011 (*p* < 0.001). *Shigella* infections increased during this period, with a precipitous rise from 8.3% in 2010 to 12.1% in 2011, but the difference was not statistically significant (**Figure 1A**). A total of 365 *Salmonella* and 218 *Shigella* isolates were collected. The five most commonly isolated *Salmonella* serovars from ill children remained constant; *S*. Typhimurium was consistently the top serovar (19–21%) followed by *S*. Agona (9%), *S*. Muenchen (6–7%), *S*. Muenster (6–11%), and *S*. Enteritidis (5–7%). The *S*. Typhi isolate was susceptible to all tested antibiotics. Similarly, there was no change in the distribution of *Shigella* species; *S. flexneri *was predominant (68–76%), followed by *S. sonnei* (16–17%), *S. boydii* (5–12%), and *S. dysenteriae* (2%). *S. dysenteriae* strains belonged to type 3 (two isolates) and type 2 (two isolates).

### CLINICAL PRESENTATION

Prospectively obtained clinical data was available for 112 patients with *Shigella* and 118 patients with *Salmonella*. Compared to children with *Salmonella*, those with *Shigella* had more fever ≥ 38.5°C (36 vs 17%, *p* = 0.001, OR = 2.7, 95% CI = 1.4–5.3), bloody stools (59 vs 36%, *p* < 0.001, OR = 2.6, 95% CI = 1.5–4.6), dehydration (27 vs 15%, *p* = 0.031, OR = 2.0, 95% CI = 1.0–4.1), and almost four times as many seizures (11 vs 3%, *p* = 0.03, OR = 3.4, 95% CI = 0.98–13.0; **Figure 1B**).

### ANTIMICROBIAL RESISTANCE

**FIGURE 2:**
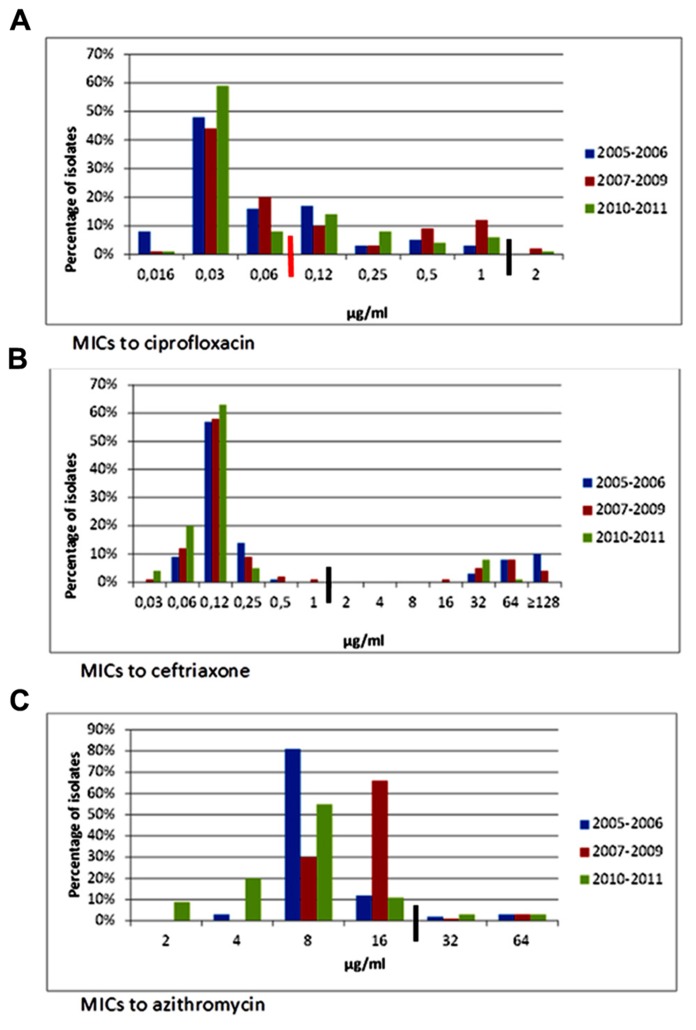
**Minimum inhibitory concentrations to ciprofloxacin, ceftriaxone, and azithromycin in *Salmonella* isolates from ill children at a state referral hospital in Yucatan, Mexico, 2005–2011.** Thick black lines indicate breakpoints for resistance. Number of isolates for each period was as follows: 2005–2006, *n* = 147; 2007–2009, *n* = 138, and 2010–2011, *n* = 80. **(A)** Ciprofloxacin MICs. Reduced susceptibility to ciprofloxacin in *Salmonella* rose from 28% in 2005–2006 to 33% in 2010–2011. Non-susceptibility (MIC = 2) first emerged in 2007 in a *S*. Anatum isolate and later appeared in *S*. Typhimurium multidrug-resistant, cephalosporin-resistant isolates. Black line is resistance breakpoint for isolates from stools; red line is breakpoint for systemic infections. CLSI breakpoints for susceptibility and resistance in isolates from stools are 1 and 4 μg/ml, respectively. Breakpoints for susceptibility and resistance in isolates from systemic infections are 0.06 and 1 μg/ml, respectively. **(B)** Ceftriaxone MICs. Resistance to ceftriaxone and other extended-spectrum cephalosporins significantly decreased from 19.7% during 2005–2006 to 8.8% in 2010–2011. *Salmonella* isolates were distributed into two distinct populations, one which was susceptible and the other resistant to ceftriaxone. CLSI breakpoints for susceptibility and resistance are 1 and 4 μg/ml, respectively. **(C)** Azithromycin MICs. No significant changes in azithromycin MICs were noted. Suggested breakpoints for susceptibility and resistance are 16 and 64 μg/ml, respectively (Sjolund-Karlsson et al., 2011).

Ampicillin resistance in *Salmonella* decreased from 43% in 2005 to 16% in 2011 (*p* < 0.001), while trimethoprim–sulfamethoxazole resistance decreased from 44% to 26% (*p* = 0.014). Likewise, resistance to ceftriaxone and other ESC, most commonly observed in *S*. Typhimurium, decreased from 27% to 10% (*p* = 0.009). Trends for the different time periods throughout the study are shown on **Table 2** and **Figures 2A–C**. MIC_50_ and MIC_90_ values for the whole study period were, respectively, 0.03 and 0.5 μg/ml for ciprofloxacin, 0.125 and 64 μg/ml for ceftriaxone, and 8 and 16 μg/ml for azithromycin.

**Table 2:** Antimicrobial susceptibility in *Salmonella* isolates from ill children inYucatan, Mexico, 2005–2011. Percentage of resistance^*^.

Year of isolation	AMP^†^	CRO^‡^	CAZ^‡^	AZT	CIP	NAL	CHL	FZD	GEN	STX^§^	TET
2005–2006 (*n* = 147)	33.3	19.7	19.7	4.8	0	29.2	33.4	30.8	12.2	38.1	52.4
2007–2009 (*n* = 138)	25.4	18.1	18.1	3.6	2.2	37.7	31.9	28.4	9.4	34.1	45.7
2010–2011 (*n* = 80)	20.0	8.8	8.8	5.0	1.2	31.3	33.8	28.7	7.5	28.8	42.5

*AMP, ampicillin; AZT, azithromycin; CAZ, ceftazidime; CHL, chloramphenicol; CIP, ciprofloxacin; CRO, ceftriaxone; FZD, furazolidone; GEN, gentamicin; NAL, nalidixic acid; STX, trimethoprim–sulfamethoxazole; TET, tetracycline.*

* Includes resistant and intermediate strains.

† Difference from 2005 to 2011: *p* < 0.001.

‡ Difference from 2005 to 2011: *p* = 0.009.

§ Difference from 2005 to 2011: *p* = 0.014.

Reduced susceptibility to ciprofloxacin (MIC ≥ 0.12 and ≤ 1 μg/ml) was found in 113 *Salmonella* isolates evenly distributed throughout the study period; 77 of these (68%) were resistant to nalidixic acid, and was most commonly seen in *S*. Typhimurium (34%), *S*. Enteritidis (16%), *S*. Albany (9%), *S*. Muenster (6%), *S*. Muenchen (5%), and *S*. Reading (5%).

The percentage of isolates with reduced susceptibility to ciprofloxacin rose from 30% in 2005 to 41% in 2011 (*p* < 0.001). The number of isolates with MIC ≥ 1 μg/ml, however, peaked during 2007–2009 (14%) and then decreased in 2010–2011 (7%), although the difference was not significant (**Figure 2A**). Isolates with ciprofloxacin MIC = 2 μg/ml were first detected in 2007 in *S*. Anatum, and subsequently, in two MDR, ESC-resistant *S*. Typhimurium strains (2008 and 2011), and one *S*. Enteritidis strain (2009).

Sixty-one ceftriaxone and ceftazidime resistant isolates were uniformly resistant to cefotaxime, cefoxitin, aztreonam, and amoxicillin/clavulanic acid; 52% were resistant to piperacillin/tazobactam. All isolates were susceptible to cefepime, imipenem, and meropenem, and were inhibited by cefoxitin in the presence of boronic acid. Before 2005, ESC-resistance was limited to *S*. Typhimurium; it was subsequently detected in *S*. Havana (2005), *S*. Anatum (2007), *S*. Agona (2008), *S*. Enteritidis (2009), and *S*. Weltevreden (2011).

**FIGURE 3:**
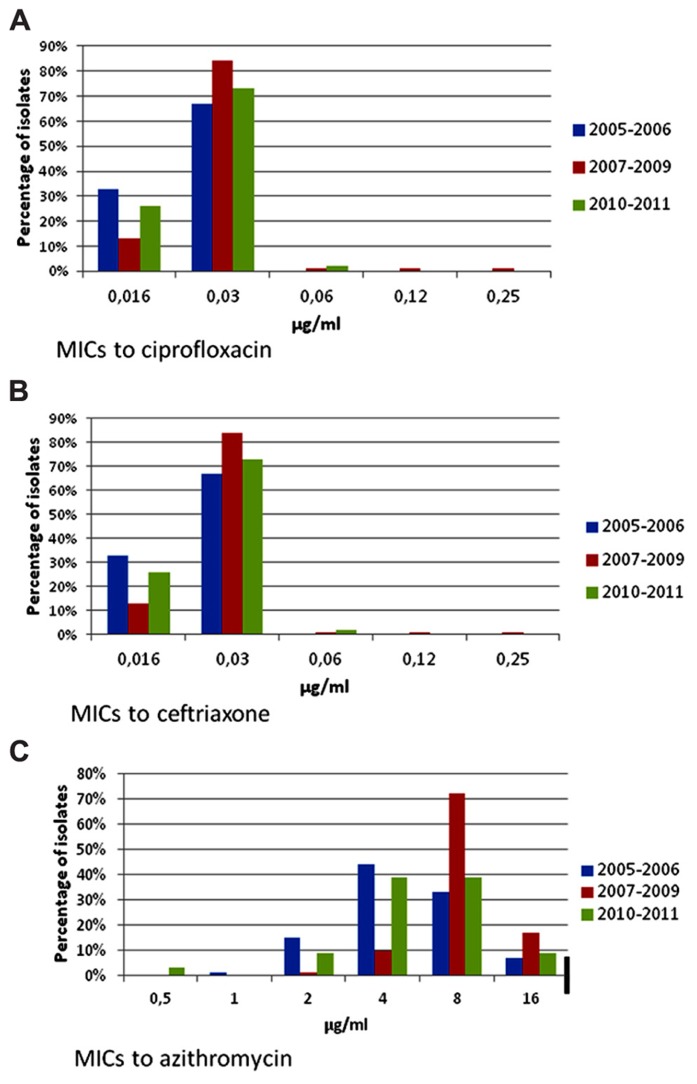
**Minimum inhibitory concentrations to ciprofloxacin, ceftriaxone, and azithromycin in *Shigella* isolates from children with gastroenteritis at a state referral hospital inYucatan, Mexico, 2005–2011.** Thick black lines indicate breakpoints for resistance. Number of isolates for each period was as follows: 2005–2006, *n* = 73; 2007–2009, *n* = 79 and 2010–2011, *n* = 66. **(A)** Ciprofloxacin MICs. Isolates were fully susceptible to ciprofloxacin throughout the study. Two isolates had ciprofloxacin MICs ≥ 0.12 μg/ml, only one of which was resistant to nalidixic acid. CLSI breakpoints for susceptibility and resistance are 1 and 4 μg/ml, respectively. **(B)** Ceftriaxone MICs. All isolates remained fully susceptible to ceftriaxone with no changes over the study period. CLSI breakpoints for susceptibility and resistance are 1 and 4 μg/ml, respectively. **(C)** Azithromycin MICs. Suggested breakpoints are the same as those proposed for *Salmonella*. Although isolates were apparently susceptible to azithromycin throughout the study period, 7–17% of these had an MIC = 16.

*Shigella* isolates were frequently resistant to tetracycline, ampicillin, furazolidone, and trimethoprim–sulfamethoxazole (**Table 3**); all isolates were susceptible to ciprofloxacin, ceftriaxone, and azithromycin. Only one isolate was resistant to nalidixic acid. Resistance to furazolidone decreased from 56.1% in 2005 to 27.1% in 2011 (*p* < 0.001). There were few changes in the MIC distributions for azithromycin, ceftriaxone, and ciprofloxacin (**Figures 3A–C**), and no significant resistance trends during the different time periods (**Table 3**). MIC_50_ and MIC_90_ values for the whole study period were, respectively, 0.03 and 0.03 μg/ml for ciprofloxacin, 0.06 and 0.06 μg/ml for ceftriaxone, and 8 and 16 μg/ml for azithromycin.

**Table 3:** Antimicrobial susceptibility in *Shigella* isolates from ill children inYucatan, Mexico, 2005–2011. Percentage of resistance^*^.

Year of isolation	AMP	CRO	AZT	CIP	NAL	CHL	FZD^†^	STX	TET
2005–2006 (*n* = 73)	39.8	0	0	0	0	16.4	57.5	57.5	83.6
2007–2009 (*n* = 79)	44.3	0	0	0	1.3	24.1	25.4	59.5	92.4
2010–2011 (*n* = 66)	34.8	0	0	0	0	12.1	25.8	59.0	93.9

*AMP, ampicillin; AZT, azithromycin; CAZ, ceftazidime; CHL, chloramphenicol; CIP, ciprofloxacin; CRO, ceftriaxone; FZD, furazolidone; GEN, gentamicin; NAL, nalidixic acid, STX, trimethoprim–sulfamethoxazole; TET, tetracycline.*

* Includes resistant and intermediate strains.

† Difference from 2005 to 2011: *p* < 0.001.

### PREVALENCE OF *qnr* AND BETA-LACTAMASE GENES

Of the 117 *Salmonella* isolates with a ciprofloxacin MIC between 0.125 and 2 μg/ml, 42 (36%) harbored *qnr* genes. Four (3%) carried *qnrA* genes and 38 (32%) carried *qnrB* genes. The number of isolates with *qnr* genes peaked in 2007 (29%) and decreased to 10% by 2011 (*p* < 0.001). The *qnrA* genes, found in two serovars, were 99% identical to *qnrA1* gene variants. *qnrB* genes, 99% identical to *qnrB19* variants, were distributed in 11 different serovars (**Table 4**). Both Qnr determinants presented 100% identity to the originally reported amino acid sequences. All 61 ESC-resistant *Salmonella* isolates carried a *bla*_CMY_ gene. The sequences of all PCR products were 99% identical to the prototype *bla*_CMY-2_ gene and had 100% identity to the those obtained for *bla*_CMY-2_ genes found in *S*. Typhimurium strains from Yucatan and three other states in Mexico (Wiesner et al., 2009). Only one isolate also harbored a TEM-1 gene; none harbored SHV, CTX, or OXA genes.

**Table 4:** Serotype distribution and antimicrobial susceptibility of *Salmonella* isolates positive for the *qnr* gene, Yucatan, Mexico, 2005–2011.

*Qnr* variant	ID number	Serotype	Year of isolation	Resistance phenotypes to non-quinolone antibiotics	Ciprofloxacin MIC (μg/ml)	Nalidixic acid MIC (μg/ml)
A1	yuhs10-35	Enteritidis	2010	Nal, Fzd	1	128
	yuhs05-13	Havana	2005	Amp, Sxt, Tet	0.5	32
	yuhs05-87	Havana	2005	Amp, Sxt, Tet, Nal	0.5	16
	yuhs05-106	Havana	2005	Amp, Sxt, Tet, Cro	0.5	16
B19	yuhs07-5	Adelaide	2007	Nal, Fzd	0.12	32
	yuhs07-44	Adelaide	2007	Nal	0.5	64
	yuhs07-71	Adelaide	2007	Nal	1	64
	yuhs08-31	Adelaide	2008	Nal	1	64
	yuhs07-40	Agona	2007	Nal	0.5	32
	yuhs08-34	Agona	2008	Nal	1	64
	yuhs07-38	Anatum	2007	Amp, Sxt, Tet, Nal, Cro	1	64
	yuhs07-53	Anatum	2007	Amp, Sxt, Tet, Nal	2	128
	yuhs09-10	Derby	2009	Tet, Nal	0.5	64
	yuhs07-61	Enteritidis	2007	Nal, Fzd	1	64
	yuhs08-57	Enteritidis	2008	Nal, Fzd	1	64
	yuhs08-59	Enteritidis	2008	Nal, Fzd	1	64
	yuhs09-1	Enteritidis	2009	Nal, Fzd	2	64
	yuhs09-7	Enteritidis	2009	Nal, Fzd	1	64
	yuhs11-43	Enteritidis	2011	Nal, Fzd	1	64
	yuhs07-45	Havana	2007	Sxt, Tet, Chl	0.5	16
	yuhs07-28	Muenchen	2007	Nal	1	32
B19	yuhs07-41	Muenchen	2007	Nal	1	32
	yuhs08-8-1	Muenchen	2008	Nal	0.5	32
	yuhs08-10	Muenchen	2008	Sxt, Nal	0.5	32
	yuhs08-30-2	Muenchen	2008	Nal	0.5	32
	yuhs11-33	Muenchen	2011	Nal	0.5	32
	yuhs05-97	Muenster	2005	Amp, Sxt, Tet, Nal, Chl, Fzd	0.12	32
	yuhs07-74-2	Muenster	2007	Sxt, Tet, Nal, Chl	1	32
	yuhs07-75	Muenster	2007	Sxt, Tet, Nal, Chl	1	32
	yuhs10-7	Muenster	2010	Sxt, Tet, Chl, Nal	1	32
	yuhs11-10	Muenster	2011	Sxt, Tet, Chl, Nal	1	32
	yuhs06-59	Reading	2006	Tet, Nal	1	32
	yuhs06-60	Reading	2006	Tet, Nal	1	32
	yuhs06-65	Reading	2006	Tet, Nal	1	32
	yuhs07-78	Typhimurium	2007	Amp, Tet, Nal, Cro, Chl	1	32
	yuhs07-79	Typhimurium	2007	Amp, Tet, Nal, Cro, Chl	1	32
	yuhs07-9	Typhimurium	2007	Amp, Sxt, Tet, Nal, Cro, Chl	1	32
	yuhs08-60	Typhimurium	2008	Amp, Sxt, Tet, Nal, Cro, Chl, Fzd, Gen	2	64
	yuhs10-16	Typhimurium	2010	Amp, Sxt, Tet, Nal, Cro, Chl	0.25	32
	yuhs11-28	Typhimurium	2011	Amp, Sxt, Tet, Nal, Cro, Chl	2	64
	yuhs11-47	Typhimurium	2011	Tet, Chl, Nal	1	32
	yuhs07-20	Untypable	2007	Nal	1	32

*AMP, ampicillin; CHL, chloramphenicol; CRO, ceftriaxone; FZD, furazolidone; GEN, gentamicin; NAL, nalidixic acid; STX, trimethoprim–sulfamethoxazole; TET, tetracycline.*

## DISCUSSION

Illness from *Salmonella* has decreased in the Yucatan Peninsula while severe *Shigella* infections have increased among children with gastroenteritis requiring hospitalization. There is a high frequency of resistance to ampicillin and trimethoprim–sulfamethoxazole in *Shigella* isolates, but these remain uniformly susceptible to ESC, ciprofloxacin, and azithromycin. While *Shigella* resistance to clinically important antibiotics over the last 7 years remained basically unchanged, resistance to most of these, with the exception of ciprofloxacin, declined in *Salmonella*. Resistance to ESC continues to be prevalent among *Salmonella* isolates, and is mediated by the *bla*_CMY-2_ gene. *qnrB* genes are widely distributed among our *Salmonella* population, but their overall frequency appear to be decreasing in recent years.

The increase in shigellosis in our pediatric population is a major public health concern, as a high proportion of our patients presented bloody diarrhea, high fever, and dehydration. The frequency of seizures (11%), moreover, is much higher than that reported in a recent study in Asia (3%; von Seidlein et al., 2006). This could be explained by the higher proportion of *S. flexneri* infections at our center and by the fact that our study was confined to children with diarrhea sufficiently severe to seek medical care at a hospital emergency room.

Our *Shigella* strains were frequently resistant to trimethoprim–sulfamethoxazole and ampicillin, the first line empirical therapy for bloody diarrhea at our public hospitals. Although all strains were susceptible to azithromycin, ESCs, and ciprofloxacin, none of these antimicrobials are provided in oral form by our government healthcare system, which services 70% of the population. Consequently, patients with therapeutic failure ultimately required hospitalization with parenteral ceftriaxone or out-of-pocket purchase of oral azithromycin or cefixime, escalating costs for both the public health sector and families.

Although the increase in *Shigella* gastroenteritis was not statistically significant, we believe, nonetheless, that our findings are epidemiologically and clinically significant, as the prevalence of *Shigella* gastroenteritis had never, during the last decade, reached 12%. It has remained at this level during 2012 and 2013 – a worrisome trend that warrants close scrutiny by public health authorities.

Our results concur with those of other investigators who challenge the widely accepted notion of a worldwide decline in shigellosis. Recent studies using active or passive surveillance have detected high incidence rates of shigellosis in Peru, Thailand, and China (Chompook et al., 2005; Kosek et al., 2008; Xia et al., 2011). Unlike Southeast Asia (von Seidlein et al., 2006; Vinh et al., 2009), where the dominant species has shifted from *S. flexneri* to *S. sonnei*, the former continues to be the major culprit of severe shigellosis in Yucatan, as is the case in Peru and China.

The troubling recent rise in shigellosis could well be linked to the current economy. Between 2008 and 2010, the number of people below the poverty line in Yucatan increased by 35,000 (CONEVAL, 2012). Several independent studies have shown that the risk factors for *S. flexneri* infection include poverty and overcrowded living conditions (Admoni et al., 1995; Kosek et al., 2008). Interestingly, a massive spike in shigellosis has recently been reported in the USA (Andrews, 2012).

In addition to a significant decrease in *Salmonella* gastroenteritis, isolates showed a downward trend in resistance to almost all antibiotics, which was unrelated to changes in serovar distribution. The spread of ESC resistance to other serovars suggests that the *bla*_CMY-2_ gene previously found in MDR *S*. Typhimurium isolates in Yucatan (Zaidi et al., 2007) may have spread, as noted in other reports (Arlet et al., 2006), through horizontal transfer. There was remarkably little diversity of extended spectrum beta-lactamase genes as only *bla*_CMY-2_ was present in our *Salmonella* strains. *bla*_CMY-2_ also predominates in *Salmonella* isolates from humans, retail meat and animals in the USA (Zhao et al., 2009; Folster et al., 2012), although *bla*_CTX_ has been found in almost 7% of clinical turkey isolates (Wittum et al., 2012). The near absence of *bla*_SHV_, *bla*_OXA_, and *bla*_CTX_ in the *Salmonella* population of USA and Mexico strongly contrasts with reports from Europe and Asia. Recent studies from Spain (de Toro et al., 2011; Perez-Moreno et al., 2013) found an assortment of *bla* genes in human *Salmonella* isolates, including *bla*_TEM-1_, *bla*_SHV_, *bla*_CTX-M_, *bla*_PSE-1_, *bla*_OXA_, and *bla*_DHA-1_, while *bla*_CTX-M_, *bla*_TEM_, and *bla*_SHV_ were more frequently found in poultry isolates from the Netherlands (Dierikx et al., 2010). Likewise, *bla*_TEM_, *bla*_SHV_, *bla*_DHA_, *bla*_OXA_, and *bla*_CTX-M_ are common in non-typhoidal salmonellae from humans and food-animals in India, China, and Korea (Menezes et al., 2010; Tamang et al., 2011a; Yu et al., 2011a). Diversification of beta-lactamase genes depends on a myriad of factors which include, among others, selective pressures in the environment as well as enzyme efficiency (Galan et al., 2013). Unraveling the complexity of the evolutionary processes that lead to the development of resistance and transfer of resistance genes may allow us to better explain their different distributions around the world.

Reduced susceptibility to ciprofloxacin and the presence of *qnr* genes was frequent in our *Salmonella* isolates; 32% presented MICs between 0.125 and 2 μg/ml, and 12% harbored a *qnrA* or *qnrB* gene. These findings are comparable with those of other reports from Latin America in which *qnrB19* was the predominant variant. It has been detected in *Salmonella* isolates from food in Colombia (Karczmarczyk et al., 2010), and was highly prevalent in commensal enterobacteria isolated from healthy children in Peru and Bolivia (Pallecchi et al., 2009). Notably, it was also found in 32% of screened individuals living in a remote village in the Amazonas (Pallecchi et al., 2011). In contrast, a recent study conducted on *Salmonella* isolates from humans, retail meat and animals in the USA detected *qnr* genes in only 0.3% of human isolates, and none from animal sources (Sjolund-Karlsson et al., 2010). *qnr* genes are also reportedly low in China, Korea, and India (on the order of 0–3%), where *aac(6′)-lb-cr* is more commonly present (Menezes et al., 2010; Tamang et al., 2011b; Yu et al., 2011b; Chen et al., 2012; Kim et al., 2013). The origin and the mechanisms for widespread dissemination of *qnrB* genes is currently unfolding. Recent evidence points to the chromosome of *Citrobacter* spp. as the likely origin of plasmid-mediated *qnrB* (Jacoby et al., 2011). The presence of *qnrB19* genes on small colE-type plasmids (Karczmarczyk et al., 2010; Pallecchi et al., 2010), as well as their insertion on transposons located on larger plasmids (Cattoir et al., 2008; Dionisi et al., 2009) are believed to contribute to their dissemination and persistence, even in the absence of selective pressure. This could explain its ubiquity in commensal fecal flora and horizontal transfer and spread among unrelated species.

It is likely that many of our isolates with decreased susceptibility to ciprofloxacin have mutations in the quinolone resistance determining region, which was not assessed in this study. Furthermore, several of our nalidixic acid-susceptible isolates with reduced susceptibility to ciprofloxacin are likely to harbor other resistance genes such as *aac(6′)-lb-cr, qep, qnrC*, and *qnrD* that have been reported by other investigators. A more thorough search for plasmid-mediated genes, nucleotide mutations in the DNA gyrase and topoisomerase IV genes, as well as plasmid characterization will be conducted in the future.

It is noteworthy that the prevalence of *bla*_CMY-2_ and *qnr* genes decreased during the study period, possibly due to a reduced selective pressure in the regional *Salmonella* population. Although detected in several other serovars, the *bla*_CMY-2_ gene is mainly confined to our *S*. Typhimurium isolates for which swine is the major reservoir (Zaidi et al., 2007). Our *qnr* genes, on the other hand, have dispersed more widely. In the Yucatan Peninsula, all of the serovars harboring *qnr* genes are mainly associated with swine except for *S*. Enteritidis, which is poultry-associated, and *S*. Muenchen, which is more prevalent in cattle.

The encouraging decline in both the incidence and the antimicrobial resistance of *Salmonella* is likely due to interventions at the farm level. During recent years, swine producers in Yucatan have decreased the overall usage of antimicrobial compounds by the implementation of stricter biosecurity measures, greater use of vaccines, and stringent hygienic measures. Licensed veterinarians are solely responsible for determining the antibiotic regimens on farms (Jose Cervera, Asociacion Ganadera Local de Porcicultores de Merida, personal communication). Our previous molecular studies have shown that *Salmonella* strains in humans closely reflect those of locally produced food-animals, particularly swine (Zaidi et al., 2007). Improved farm management practices would thus have a direct impact on zoonotic pathogens such as *Salmonella*, but would be unlikely to have any effect on strictly human pathogens such as *Shigella*, an assumption supported by our study results. A comprehensive study in Denmark (World Health Organization [WHO], 2003) showed that an overall reduction in usage of antimicrobial compounds in food-animals was associated with decreased resistance in foodborne pathogens.

The epidemiology of *Salmonella* and *Shigella* in the Yucatan Peninsula, as in other regions of the world, is evolving. Typhoid fever and *Shigella dysenteriae* type 1infections, the major scourges four decades ago, are now rare. The new challenges, as evidenced from this study, are MDR, ESC-resistant *Salmonella* with rising resistance to ciprofloxacin and severe *S. flexneri* gastroenteritis. Our findings underscore the need for greater efforts to preserve critically important antibiotic classes such as ESC and fluoroquinolones. The agricultural sector in Mexico has taken important steps to reduce the burden of salmonellosis. Comparable action to ameliorate the impact of shigellosis is urgently required. If worsening socioeconomic conditions are a major determinant of this increase, action by health authorities alone will not suffice. Policy makers must take account of the full consequences of social inequality and act accordingly (Stiglitz, 2012).

## Conflict of Interest Statement

The authors declare that the research was conducted in the absence of any commercial or financial relationships that could be construed as a potential conflict of interest.
